# Obesity and sudden cardiac death: Prevalence, pathogenesis, prevention and intervention

**DOI:** 10.3389/fcell.2022.1044923

**Published:** 2022-12-02

**Authors:** Yan Yao, Jia Xue, Bing Li

**Affiliations:** Department of Cardiology, Beijing Anzhen Hospital, Capital Medical University, Beijing, China

**Keywords:** obesity, sudden cardiac death, remodeling, metabolic dysfunction, weight management

## Abstract

Obesity and sudden cardiac death (SCD) share common risk factors. Obesity, in and of itself, can result in the development of SCD. Numerous epidemiologic and clinical studies have demonstrated the close relationships between obesity and SCD, however, the underlying mechanisms remain incompletely understood. Various evidences support the significance of excess adiposity in determining the risk of SCD, including anatomical remodeling, electrical remodeling, metabolic dysfunction, autonomic imbalance. Weight reduction has improved obesity related comorbidities, and reversed abnormal cardiac remodeling. Indeed, it is still unknown whether weight loss contributes to decreased risk of SCD. Further high-quality, prospective trials are needed to strengthen our understanding on weight management and SCD.

## Introduction

Obesity, characterized by an increase in adipose mass and dysregulation of adipose cells, is becoming a progressively prevalent metabolic disorder. According to the classification of the body mass index (BMI), overweight is defined as a BMI of 25 ≤ BMI<30 kg/m^2^, and obesity as a BMI of ≥30 kg/m^2^ (Jensen et al., 2014). A number of studies have found that obesity is associated with extensive social, economic and clinical implications, resulting in an increased risk of cardiovascular disease and mortality (Powell-Wiley et al., 2021).

**FIGURE 1:**
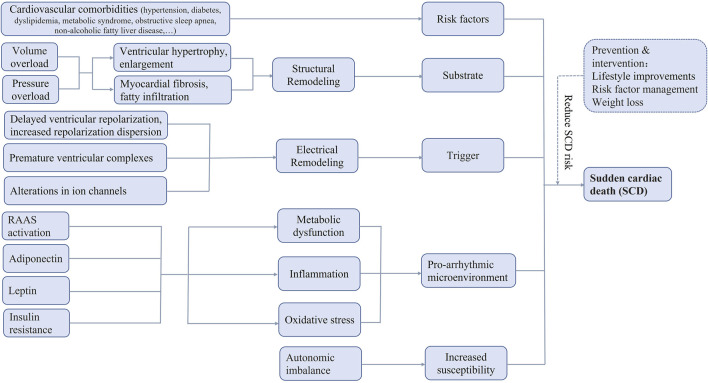
Possible pathophysiologic mechanisms of sudden cardiac death in obesity. RAAS, renin-angiotensin-aldosterone system, SCD, sudden cardiac death.

The most widely accepted definition of sudden cardiac death (SCD) is unexpected death attributable to cardiovascular causes occurring within 1 h of the onset of acute symptoms, in an individual with or without known pre-existing heart diseases. SCD is responsible for up to 20% of all-cause deaths and up to 50% of all cardiovascular deaths worldwide (Huikuri et al., 2001), constituting an increasing global health burden in Figure 1.

Traditional cardiovascular risk factors and obesity often coexist, such as aging, hypertension, coronary heart disease, diabetes mellitus. Other risk factors include metabolic syndrome, obstructive sleep apnea, non-alcoholic fatty liver disease, and possibly male gender, etc (Plourde et al., 2014). These comorbidities potentially contribute to an increased risk of SCD for obese patients, however, obesity, in and of itself, can lead to the development of SCD directly (Jensen et al., 2014). Obese patients have been documented to have higher rates of arrhythmia and SCD in the absence of cardiac dysfunction (Bastien et al., 2014).

## Prevalence

The close relationships between obesity and the risk of SCD have been observed in numerous epidemiologic and clinical studies. In the 4th century, Hippocrates found that “sudden death is more common in those who are naturally fat than in the lean.”

A series of findings from the Framingham Heart Study confirmed the association between obesity and SCD. In a 1975 study of 4,120 men aged 45–74 years, the risk of SCD was particularly marked in grossly overweight men and increased progressively with the degree of overweight, furthermore, obesity had a significant, independent contribution to SCD with a follow-up for 16 years (Kannel et al., 1975). In a 1983 study of 5,209 participants with 26-year follow-up, there was a much stronger gradient of risk for sudden death with increasing weight in both men and women (Hubert et al., 1983). Among individuals older than 50 years, the heaviest group experienced twice the risk of SCD compared with the leanest group in men, and the risk was increased 6.8-fold in obese women (Hubert et al., 1983).

In the multicentre, prospective, ARIC study of 14,941 participants over 12 years of follow-up, general obesity was associated with increased risk of SCD in middle-aged, non-smoking individuals (Adabag et al., 2015). Higher waist-to-hip ratio, a marker of central obesity, was reported to be a stronger factor for SCD than BMI or waist circumference, suggesting the important role of abdominal adiposity in SCD(Adabag et al., 2015). And larger sagittal abdominal diameter was associated with a particularly increased risk of SCD in asymptomatic French middle-aged men, independent of BMI and other cardiovascular risk factors (Empana et al., 2004).

In the prospective Nurses’ Health Study, the risk of SCD was increased 1.46-fold for BMI 25.0–29.9, 1.46-fold for BMI 30.0–34.9, and 2.18-fold for BMI ≥35.0, higher BMI was significantly associated with greater SCD risk after adjustment for confounders (Chiuve et al., 2015). In a cohort of 10,543 middle-aged Finnish population, the overweight and obese subjects were at increased risk for SCD with hazard ratios 1.33 and 1.79, respectively (Eranti et al., 2016). A meta-analysis of 14 prospective studies consisting of 406,079 participants, found that every 5-unit increment in BMI conferred a 16% higher risk of SCD, and there was a J-shaped association between BMI and SCD with the normal weight range patients at the lowest risk (Aune et al., 2018).

Evidences from epidemiological studies showed greater BMI or obesity had strong associations with SCD independent of established risk factors, clinical evidences also supported these findings. A recent study assessed the causes of SCD in an autopsy series of nonischemic SCD victims, demonstrating that obesity related cardiomyopathy was the most common reason and responsible for 23.7% of the cases (Hookana et al., 2011). Besides, even though patients with nonischemic SCD had higher BMI, they also had a lower incidence of hypertension, diabetes and hypercholesterolemia (Hookana et al., 2011). In the Multicenter Automatic Defibrillator Implantation Trial II, obesity increased the risk of appropriate therapy for ventricular tachyarrhythmia/ventricular fibrillation by 64% in patients with ischemic cardiomyopathy (Pietrasik et al., 2007; Sabbag et al., 2016). These suggested the great influence of obesity on the risk of lethal cardiac arrhythmias and SCD.

## Structural remodeling

There are various evidences to support the significance of excess adiposity in determining the risk of SCD, including anatomical factors, electrical factors, metabolic factors, etc. Both mild obesity and severe obesity have been reported to be associated with greater risk of ventricular tachycardia/ventricular fibrillation, highlighting its role in the formation of arrhythmogenic substrate (Sabbag et al., 2016). In obese hearts, a series of hemodynamic disorders and abnormal neuroendocrine activation finally result in the ventricular remodeling, including left ventricular hypertrophy, left ventricle enlargement, consequential systolic and diastolic dysfunction, together with myocardial hypertrophy, fibrosis, focal myocardial disarray, fatty infiltration, and myocardial steatosis at the pathological level (Abel et al., 2008; Russo et al., 2011). All these changes may contribute to the increased propensity to ventricular arrhythmias and SCD in obese individuals.

### Left ventricular hypertrophy and enlargement

Left ventricular hypertrophy and enlargement are common in obese patients. In early post-mortem and biopsy studies, it demonstrated that severe and long-standing obesity, particularly when accompanied by hypertension and congestive heart failure, was associated with left ventricular dilation and hypertrophy and, to a lesser extent, right ventricular dilation and hypertrophy (Harmancey et al., 2008; Alpert et al., 2014). Obesity could predispose to heart failure of both ventricles. Post-mortem studies on obese subjects consisted largely of morbidly obese patients, it might overlook cardiac morphology of asymptomatic and symptomatic persons with various degrees of obesity. Then, several echocardiographic studies found a significant positive correlation between the severity of obesity and left ventricular mass, and left ventricular chamber size even after adjustment for height. In a large cohort study enrolling 11,792 obese patients with preserved ejection fraction, there was a prevalence of 49% for abnormal left ventricular geometry including concentric remodeling, eccentric and concentric hypertrophy (Lavie et al., 2007). And abnormal left ventricular geometry occurred much more commonly in obese than in nonobese patients (Patel et al., 2014). These structural abnormalities in left ventricle *per se* give rise to increased ventricular arrhythmias.

Obesity cardiomyopathy occurs when these cardiac structural and hemodynamic changes result in congestive heart failure (Harmancey et al., 2008; Ren et al., 2021). Obesity cardiomyopathy usually happens to individuals with severe and long-standing obesity. Left ventricular hypertrophy and diastolic dysfunction are early manifestations in the disease course, left ventricular systolic function becomes decompensated and finally symptomatic heart failure occurs (Alpert et al., 2014; Plourde et al., 2014). There are always two causes of death for obesity cardiomyopathy patients, one is progressive congestive heart failure, and the other is SCD(Plourde et al., 2014).

Volume and pressure overload might take part in the process of left ventricular remodeling for obese subjects. Obesity is associated with increased total blood volume and cardiac output induced by the high metabolic activity of excessive adiposity, which will lead to volume overload (Alpert, 2001). Besides, hypertension is so common in obese populations, chronic cardiac pressure overload caused by elevated systemic blood pressure may partly contribute to left ventricular hypertrophy (Cavalera, 2014)**.** Volume and pressure overload have different effects on the cardiomyocytes, volume overload leads to chamber dilation and matrix degradation, whereas pressure load induces concentric hypertrophy, increased collagen deposition, and diastolic dysfunction (Ulasova et al., 2011; Cavalera, 2014). It is difficult to evaluate the relative contribution of these pathophysiologic changes during the remodeling process in obese patients. Pulmonary hypertension and coexisting obstructive sleep apnea/obesity hypoventilation syndrome will increase right ventricular wall stress, finally do damage to right ventricular structure and function (Jordan et al., 2014). Abnormal neuroendocrine activation ensues further bringing in progression of both diastolic and systolic dysfunction.

### Myocardial fibrosis and fatty infiltration

Myocardial fibrosis has been documented in most animal models of obesity. db/db mice with a mutation which caused a truncated long-form of the leptin receptor, had a hyperphagic appetite, rapidly significant obesity and diabetes during the first months of life. At the age of 4–6 months, cardiac interstitial fibrosis and diastolic dysfunction could be easily observed in db/db mice (Sloan et al., 2011; Gonzalez-Quesada et al., 2013). Obese hyperinsulinemic Zucker rats also showed perivascular cardiac fibrosis, associated with increased left ventricular mass and cardiac hypertrophy (Fredersdorf et al., 2004). Not only genetically edited obese mice but also diet-induced obese mice, could develop the phenotype of cardiac fibrosis. In male C57BL6J mice fed a high-fat high-carbohydrate diet for 6 months, histological and echocardiographic findings demonstrated progressive left ventricular hypertrophy, interstitial fibrosis, and diastolic dysfunction (Qin et al., 2012). Obesity might also enhance the effects of other fibrotic changes. In models of hypertensive fibrosis, obesity increased cardiac hypertrophy, collagen deposition and extracellular matrix remodeling (van Bilsen et al., 2014). In mice with reperfused myocardial infarction, both genetic and diet-induced obesity accentuated post-infarction ventricular dilation (Thakker et al., 2006).

Fatty infiltration is prevalent in the obese heart. Excessive fat might be widely visible on the epicardium, the pericardium and the perivascular tissues. It could also infiltrate into the myocardium (Plourde et al., 2014). Increased volume of fat might secrete inflammatory cytokines and promote inflammatory responses within the myocardium *via* an autocrine or paracrine manner. Myocardial steatosis is another potential mechanism in obesity cardiomyopathy at the pathological level.

## Electrical remodeling

Alterations in cardiac cellular electrophysiology due to obesity are complex and may include delayed ventricular repolarization, increased repolarization dispersion, premature ventricular complexes, and alterations in ion channels. Cardiac structural remodeling provides the arrhythmogenic substrate for obesity related SCD, while electrical remodeling acts as a direct contributor to SCD.

### Delayed ventricular repolarization and increased repolarization dispersion

Prolonged and heterogeneous ventricular repolarization is the characteristic of obesity-associated electrical abnormalities (Mukerji et al., 2012a). QT interval and QT interval corrected to the heart rate (QTc) represent ventricular repolarization duration, and prolonged QT interval is always considered as the increased risk of fatal ventricular arrhythmias including ventricular tachycardia and ventricular fibrillation (Omran et al., 2018). The Bazett formula is most commonly used that correlate QTc to SCD. A QTc of 440 ms has been widely identified as a cut-off value indicative of increased SCD risk. QT dispersion is the difference between the longest and shortest QT interval, and is thought to reflect heterogeneity of repolarization (Omran et al., 2018).

Cohort studies showed that obese patients were more likely to have prolonged QT intervals than those with normal weight (Mukerji et al., 2012a). In the EURODIAB Type 1 Complications Study, the overall prevalence of QT prolongation was 16%, that was 11% in men, and 21% in women (Veglio et al., 1999). Obvious QTc prolongation and enhanced QT dispersion could be even evidenced at an early age in obese children. A meta-analysis also found that QT/QTc and QT dispersion in obese and overweight populations was much longer than in those with normal weight (Omran et al., 2016). QRS fragmentation, a surrogate for heterogeneous conduction, was also manifested in obesity and obesity-mediated SCD(Narayanan et al., 2015). And QRS fragmentation was confirmed as an independent predictor of SCD.

Among obese patients, those who had left ventricular hypertrophy showed much longer QTc interval. Left ventricular hypertrophy was an indicator of the onset of cardiac decompensation, and the development of electrical abnormalities was involved as a consequence (Russo et al., 2011). Obesity cardiomyopathy was taken as the key culprit to the delayed ventricular repolarization and increased repolarization dispersion, which facilitated the occurrence of reentrant ventricular arrhythmias (Ren et al., 2021).

### Premature ventricular complexes

In the obese population, left ventricular dilation was associated with premature ventricular contractions, since the structural abnormalities always brought in the changes in electrical properties. Premature ventricular beats, despite generally unharmful, conferred a higher risk of ventricular tachyarrhythmias and mortality in the general population, even if the overall burden was low. Continuous ECG monitoring demonstrated obese subjects had increased frequency of ventricular beats (Messerli et al., 1987). When the prolonged QTc and increased dispersion concurred with premature ventricular beats, it might result in the development of ventricular arrhythmias triggered by early or delayed after-depolarization (Bikkina et al., 1993).

Epicardial adipose tissue was found to have close relationships with high probability of premature ventricular beats, ventricular tachyarrhythmias, and all-cause mortality from SCD(Wu et al., 2015). The intramyocardial adiposity and heterogeneous conduction led to abnormal electrophysiological properties, and higher propensity for ventricular tachycardia. In addition, epicardial fatty infiltrations, myocardial steatosis, and subsequent cardiac fibrosis might form reentrant circuits for lethal arrhythmias (Pouliopoulos et al., 2013).

Late potentials are considered to represent abnormal conduction in the myocardium, providing a possible site for re-entry and then ventricular tachyarrhythmias initiation (Plourde et al., 2014). A filtered QRS duration >114 ms in signal averaged electrocardiography is thought to be the optimal value associated with arrhythmic risk (Goldberger et al., 2008). Late potentials were documented in obese patients, there was a positive relationship between BMI and the number of late potentials, indicating an increased susceptibility to ventricular tachyarrhythmias and SCD(Lalani et al., 2000).

### Alterations in ion channels

It is well-known that QTc prolongation, QT dispersion and premature ventricular beats are common manifestations of electrocardiogram, the fundamental cellular electrophysiological mechanisms are attribute to the expression and function changes of various cardiac ion channels, including voltage-dependent potassium channels, sodium channels and calcium channels, and perturbation of intracellular Ca^2+^ homeostasis mediated by calcium receptors and channels (Remme, 2022). Cardiac ion channels are the key mediators between obesity, abnormal repolarization and SCD.

There was a decreased expression of the inward potassium current in animal models of obesity. In mice with diet-induced obesity, the reduction of voltage-gated potassium channel resulted in action potential prolongation, subsequent clinically observed QTc prolongation, and more frequent premature ventricular contractions (Huang et al., 2013). It seemed K_ATP_ channels played an important role in prolonged QT. In drug-induced long QT syndromes in dogs and rabbits, the administration of K_ATP_ opener nicorandil led to a reduction of the action potential duration, early after depolarization, and premature ventricular beats (Biermann et al., 2011). It indicated K_ATP_ channels were inhibited in obese cardiomyocytes, and its openers could rescue the repolarization abnormalities, demonstrating a significant anti-arrhythmic effect.

Voltage-dependent L-type calcium current was reported to be decreased in obesity models, resulting in the extension of phase 2 plateau period and prolonged repolarization. The magnitude of late sodium current was increased, which caused more sodium entry into the cardiomyocyte, and increased calcium influx *via* the reverse mode of the sodium/calcium exchanger, facilitating calcium-dependent pro-arrhythmic outcome (Yang et al., 2015).

Defective calcium handling could do harm to ventricular cardiomyocytes and bring in pro-arrhythmic effects. In high-fat diet animal models, it caused abnormal oxidation of ryanodine receptor type 2 (RyR_2_, calcium release channel in sarcoplasmic reticulum), greater calcium release *via* RyR_2_, and subsequent calcium overload inducing delayed after-depolarizations. Ca^2+^/calmodulin protein kinase II (CaMKII) was thought to be vital to intracellular calcium handling. Increased activity of CaMKII took an important role in calcium-dependent pro-arrhythmic effects for inducing mitochondrial calcium overload and mitochondrial dysfunction (Hegyi et al., 2021; Remme, 2022).

These changes in ion channels were proposed as drivers of ventricular arrhythmias in obese patients. Coupled with the structural changes above, which provided the arrhythmogenic substrate, a milieu for ventricular arrhythmogenesis existed in the obese heart (Ha et al., 2022).

## Metabolic dysfunction, inflammation, oxidative stress

Obesity-related cardiomyopathy is characterized by a series of metabolic disturbances, inflammation, oxidative stress. Excess adiposity correlates with abnormal expression of many adipokines, cytokines, and hormones that have various effects on the structural and functional performances in the setting of obese heart. The imbalance of pro-inflammatory and anti-inflammatory adipokines created a chronic inflammatory state, providing a pro-arrhythmic microenvironment (Lumeng and Saltiel, 2011). Dysregulated epicardial and/or intramyocardial adipose tissue that induced macrophage infiltration could generate a profibrotic condition by cytokine release. Enhanced oxidative stress, mediated by reactive oxygen species as to mitochondrial dysfunction in adipocytes from exposure to excess free fatty acids, could give rise to cardiac fibrosis (Yang et al., 2015).

### RAAS activation

Activation of the renin-angiotensin-aldosterone system (RAAS), which always occurred in response to hemodynamic disturbances, was described in animal models of obesity. In obese salt-sensitive rats and db/db mice, it demonstrated increased expression and enhanced functional activity of angiotensin converting enzyme (ACE) and AT1 receptor (Singh et al., 2008; Takatsu et al., 2013). Activation of the RAAS would finally prompt cardiac fibrosis and remodeling through multiple signal pathways.

Pharmacological intervention with RAAS inhibitor showed positive effects on obesity related fibrosis and metabolic dysfunction. Both ACE inhibitors and AT1 blockers attenuated collagen synthesis and cardiac fibrosis, downregulated TGF-β levels, and reduced superoxide production (Zaman et al., 2001; Sloan et al., 2011; Vázquez-Medina et al., 2013). Stimulation of the mineralocorticoid receptor resulted in left ventricular hypertrophy, pro-fibrotic, and pro-inflammatory actions. The use of aldosterone antagonist could prevent these pathophysiological abnormities, and improve the survival for heart failure patients (Kosmala et al., 2013). When treated with mineralocorticoid receptors blocker, obese mice demonstrated decreased expression of proinflammatory and prothrombotic adipokines playing roles in obesity cardiomyopathy (Guo et al., 2008).

### Adiponectin

Adiponectin is an insulin-sensitizing adipokine which is exclusively produced by adipocytes. The physiological function of adiponectin includes improved insulin resistance, anti-arteriosclerosis, and anti-inflammation (Ouchi et al., 2006). The plasma level of adiponectin has good predictive value for type 2 diabetes mellitus and coronary heart disease (Ouchi et al., 2003), both of these diseases have close relationships with obesity and SCD.

The risk of SCD for obese subjects may be associated with decreased serum concentrations of adiponectin. In obese patients, there was a reduction of adiponectin which might possibly concur with other risk factors for the development of cardiomyopathy (Plourde et al., 2014). In animal models, decreased levels of adiponectin could lead to left ventricular hypertrophy through AMP kinase signaling and adrenergic receptor stimulation. Modulation of serum levels of adiponectin in knock-out mice might attenuate these adverse effects (Shibata et al., 2004).

### Leptin

Leptin, produced by white adipose tissue, is a key mediator in metabolic regulation by acting on leptin receptor. In a state of low energy or reduced body fat (such as starvation), serum leptin levels decrease significantly, conversely, when body fat increases, serum levels of leptin rise to inhibit eating and speed up metabolism. Leptin regulates energy balance and body weight through such a negative feedback mechanism (Gautron and Elmquist, 2011).

Leptin also acts as an important factor in the pathogenesis of obesity-related cardiac remodeling. In satiety-induced obesity state, increased leptin cannot result in more energy consumption, which is considered as “leptin resistance”. Serum levels of leptin was elevated, and was positively associated with left ventricular hypertrophy in obese patients (Perego et al., 2005). It seemed leptin could directly increase myocardial fat volume, induce cardiomyocyte hypertrophy (Rajapurohitam et al., 2003), which might be partly explained by leptin resistance. In addition, leptin regulates the phenotype of fibroblast, initiating a fibrogenic process (Fukui et al., 2013).

## Other factors potentially contributing to obesity-related SCD

Autonomic imbalance was associated with an increased risk of SCD in obese individuals (Bissinger, 2017). Autonomic dysfunction of ganglionic plexi was thought to be partly attribute to the chronic inflammation and oxidative stress in excessive epicardial fat (McCully et al., 2013). In the db/db mice model of type 2 diabetes mellitus, cardiac sympathetic dysfunction contributed to the increased susceptibility to ventricular arrhythmias (Jungen et al., 2019).

Obstructive sleep apnea syndrome, one of the common comorbidities of obesity, was potentially involved in the development of SCD(Gami et al., 2013). Obstructive sleep apnea syndrome was characterized by insulin resistance, endothelial dysfunction, hyoxemia, and reduced NO availability, all of these might underlie in the pathophysiological signaling pathways of SCD(Gami et al., 2005; Gami et al., 2013).

Non-alcoholic fatty liver disease (NAFLD) was prevalent in obese patients. NAFLD was always considered to be the representation of atherosclerosis in the liver, and patients with NAFLD might benefit from statin treatment. Therefore, the clinical significance of NAFLD was similar to coronary heart disease as to the risk of SCD.

## Prevention and intervention

The incidence of obesity has risen dramatically in the past decades, obesity has become an epidemic in developed countries, bringing in numerous public health problems. Now that pathophysiological mechanisms underlying obesity-related SCD are complex and not fully clarified, specific prevention and intervention strategies are limited despite many attempts. SCD is always unpredictable, making its management so challenging. Definitely, positive therapeutic treatments can also be advised (Powell-Wiley et al., 2021).

First of all, lifestyle improvements are recommended such as increased physical activity, diet optimization, smoking quitting, and active patient participation (Powell-Wiley et al., 2021). Then, it is important to deal with cardiovascular risk factors and comorbidities, including strict control on blood pressure, glucose, and lipid profiles, etc. The medication on obesity cardiomyopathy should be an effective approach for SCD prevention. As for those high-risk patients who have suffered from syncope or cardiac arrest events, an implanted cardioverter defibrillator might be considered.

There is a hot topic whether weight loss would be a useful approach for obesity-related SCD intervention. Current treatments available for weight loss include dietary control, physical exercises, diet pills, and bariatric surgery (Pathak et al., 2015). Until now, there are no adequate evidences assessing the relative benefits of these methods in reducing SCD risk. However, it seems strategies on appropriate weight-management should be considered to improve long-term outcomes for obese individuals (Ades and Savage, 2014). Recent ACC/AHA guideline has suggested aiming for reduction of approximately 5%–10% of initial weight with lifestyle improvements comprising of dietary and exercise modification, ahead of introduction of drugs or surgery options (Jensen et al., 2014; Powell-Wiley et al., 2021).

Previous animal studies and clinical trials showed weight reduction in obese patients would result in reverse structural and electrical remodeling, including reduced epicardial fat volume, improved left ventricular hypertrophy, reduced ventricular repolarization duration and heterogeneity, and improved conduction properties (Russo et al., 2007; Mukerji et al., 2012b; Al-Salameh et al., 2014; Powell-Wiley et al., 2021). With the weight loss, a series of abnormal changes would restore as to oxidative stress, adipokine profiles, inflammatory status, all of these subsequently contribute to reverse cardiac remodeling (Russo et al., 2007; Mukerji et al., 2012b; Powell-Wiley et al., 2021). Indeed, it remains to be investigated whether reverse cardiac remodeling following weight loss could reduce the risk of SCD or lethal ventricular arrhythmias due to obesity. In the Swedish Obese Subjects study, patients who underwent bariatric surgery had a non-statistically significant but higher rate of SCD(Sjöström et al., 2007). The presence of “obesity paradox” might be an underlying reason (Powell-Wiley et al., 2021). The intricate interactions between sustained weight loss and malignant arrhythmias have been poorly understood. Though the current evidences could not demonstrate a clear clinical benefit for weight reduction strategy, one might expect that significant weight loss should be protective for SCD(Powell-Wiley et al., 2021). High-quality, prospective studies are needed to address these controversies.

## Conclusion

Given the progressive rising prevalence of obesity, it will be of significant clinical importance to clarify the underlying pro-arrhythmic mechanisms in the setting of obesity. Previous studies have strengthened our understanding on the epidemiologic, structural, electrophysiological, and metabolic level. Obesity is a modifiable condition, and weight reduction has demonstrated various encouraging effects on reverse structural and electrical remodeling. Further mechanistic studies and clinical trials are needed to identify potential prognostic biomarkers, and therapeutic targets to deal with obesity and obesity-related SCD.
